# Supplementary material for: A conserved quality-control pathway that mediates degradation of unassembled ribosomal proteins
Source: eLife. 2016 Aug 23;5:e19105. doi: 10.7554/eLife.19105 (PMC5026473; doi:10.7554/eLife.19105)
Supplement: Supplementary file 1. — DOI: http://dx.doi.org/10.7554/eLife.19105.022 [file elife-19105-supp1.docx]

**Table S1. Yeast strains used in this study**

| **Strain** | **Genotype** | **Source** |
| --- | --- | --- |
| RJD1721 | (BY4741) *MAT*a *his3∆1 leu2∆0 met15∆0 ura3∆0* | - |
| RJD808 | (W303a) *MAT*a *can1-100 leu2-3,112 trp1-1 ura3-1 ade2-1 his3-11,15* | - |
| RJD6428 | BY4741 *pdr5∆::KanMX4*, p*GAL1*-*RPL13B-HHZ** | (Sung et al., 2016) |
| RJD6429 | BY4741 *pdr5∆::KanMX4*, p*GAL1-RPL26A-HHZ** | (Sung et al., 2016) |
| RJD6430 | BY4741 *pdr5∆::KanMX4*, p*GAL1-RPL34A-HHZ** | (Sung et al., 2016) |
| RJD6431 | BY4741 *pdr5∆::KanMX4*, p*GAL1-RPL36A-HHZ** | (Sung et al., 2016) |
| RJD6432 | BY4741 *pdr5∆::KanMX4*, p*GAL1-RPS17B-HHZ** | (Sung et al., 2016) |
| RJD6433 | BY4741 *pdr5∆::KanMX4*, p*GAL1-RPS18A-HHZ** | (Sung et al., 2016) |
| RJD6434 | BY4741 *pdr5∆::KanMX4*, p*GAL1-RPS24A-HHZ** | (Sung et al., 2016) |
| RJD6435 | BY4741 *pdr5∆::KanMX4*, p*GAL1-RPS24B-HHZ** | (Sung et al., 2016) |
| RJD6436 | BY4741 *pdr5∆::KanMX4*, p*GAL1-HOG1-HHZ** | (Sung et al., 2016) |
| RJD6437 | BY4741 *pdr5∆::KanMX4*, p*GAL1-HHT2-HHZ** | (Sung et al., 2016) |
| RJD6443 | BY4741, pESC(HIS3)-*P_GAL10_-RPL26A-FLAG* | (Sung et al., 2016) |
| RJD6444 | BY4741 *rpl26a∆::KanMX4 rpl26b∆::KlLEU2*, pESC(HIS3)-*P_GAL10_-RPL26A-FLAG* | (Sung et al., 2016) |
| RJD6452 | BY4741 *pdr5∆::KanMX4,* pESC(HIS3) | (Sung et al., 2016) |
| RJD6453 | BY4741 *pdr5∆::KanMX4,* pESC(HIS3)-*P_GAL10_-RPL26A-FLAG* | (Sung et al., 2016) |
| RJD6456 | BY4741 *pre9∆::KlLEU2*, pESC(HIS3) | (Sung et al., 2016) |
| RJD6459 | BY4741 *NOP56-RFP::KlURA3*, pESC(HIS3)-*P_GAL10_-RPL26A-GFP* | (Sung et al., 2016) |
| RJD6462 | BY4741 *pre9∆*::*KlLEU2,* pESC(HIS3)-*P_GAL10_-RPL26A-FLAG* | (Sung et al., 2016) |
| RJD6468 | BY4741 *tom1∆::KanMX4* | OBS* |
| RJD6470 | BY4741 *tom1∆::KanMX4*, pESC(HIS3)-*P_GAL10_-RPL26A-FLAG* | This study |
| RJD6473 | BY4741 *ubc4∆::KanMX4*, pESC(HIS3)-*P_GAL10_-RPL26A-FLAG* | This study |
| RJD6475 | BY4741 *ubc4∆::KanMX4* *ubc5∆::KlLEU2* *pre9∆::KlURA3*, pESC(HIS3)-*P_GAL10_-RPL26A-FLAG* | This study |
| RJD6476 | BY4741 *tom1C3235A::KlURA3* | This study |
| RJD6477 | BY4741 *tom1C3235A::KlURA3*, pESC(HIS3) | This study |
| RJD6478 | BY4741 *tom1C3235A::KlURA3*, pESC(HIS3)-*P_GAL10_-RPL26A-FLAG* | This study |
| RJD6481 | BY4741 *tom1C3235A*::*KlURA3* *pdr5∆*::*KlLEU2*, pESC(HIS3)-*P_GAL10_-RPL26A-FLAG* | This study |
| RJD6482 | BY4741, pESC(HIS) | This study |
| RJD6484 | BY4741 *tom1∆::KanMX4 pre9∆::KlLEU2*, pESC(HIS3)-*P_GAL10_-RPL26A-FLAG* | This study |
| RJD6485 | BY4741 *TOM1-MYC::KlURA3* | This study |
| RJD6486 | BY4741 *TOM1-HA::KlURA3* | This study |
| RJD6487 | BY4741 *TOM1-FLAG::KlURA3* | This study |
| RJD6488 | BY4741 *TOM1-GFP::KlURA3* | This study |
| RJD6489 | BY4741 *TOM1-HA::KlURA3*, pESC(HIS3)-*P_GAL10_-RPL26A-FLAG* | This study |
| RJD6491 | W303 *tom1∆::KlLEU2*, pRS314 | This study |
| RJD6492 | W303 *tom1∆::KlLEU2*, pRS314-*TOM1* | This study |
| RJD6493 | W303 *tom1∆::KlLEU2*, pRS314-*tom1C3235A* | This study |
| RJD6494 | W303 *tom1∆::KlLEU2*, pRS314-*3×HA-TOM1* | This study |
| RJD6495 | W303 *tom1∆::KlLEU2*, pRS314-*3×HA-tom1C3235A* | This study |
| RJD6496 | BY4741 *KanMX6::P_RFA1_-3×HA-TOM1* | This study |
| RJD6498 | BY4741 *KanMX6::P_RFA1_-3×HA-TOM1 pdr5∆::KlLEU2*, pESC(HIS) | This study |
| RJD6499 | BY4741 *KanMX6::P_RFA1_-3×HA-TOM1 pdr5∆::KlLEU2*, pESC(HIS)-*P_GAL10_-RPL26A-FLAG* | This study |
| RJD6500 | BY4741 *KanMX6::P_RFA1_-3×HA-tom1C3235A::KlURA3* | This study |
| RJD6502 | BY4741 *KanMX6::P_RFA1_-3×HA-tom1C3235A::KlURA3* *pdr5∆::KlLEU2*, pESC(HIS)-*P_GAL10_-RPL26A-FLAG* | This study |
| RJD6503 | BY4741 *KanMX6::P_RFA1_-3×HA-TOM1*, pESC(HIS3)-*P_GAL10_-RPL26A-FLAG* | This study |
| RJD6504 | BY4741 *KanMX6::P_RFA1_-3×HA-tom1C3235A::KlURA3*, pESC(HIS3)-*P_GAL10_-RPL26A-FLAG* | This study |
| RJD6507 | BY4741 *NOP56*-*RFP*-*KlURA3 tom1C3235A::KlLEU2*, pESC(HIS)-*P_GAL10_-RPL26A-GFP* | This study |
| RJD6508 | BY4741, pESC(HIS)-*P_GAL10_-RPL26A(R12,13E)-FLAG* | This study |
| RJD6509 | BY4741, pESC(HIS)-*P_GAL10_-RPL26A(R16E)-FLAG* | This study |
| RJD6510 | BY4741, pESC(HIS)-*P_GAL10_-RPL26A(R27,28E)-FLAG* | This study |
| RJD6511 | BY4741, pESC(HIS)-*P_GAL10_-RPL26A(R51,52E)-FLAG* | This study |
| RJD6512 | BY4741, pESC(HIS)-*P_GAL10_-RPL26A(3E)-FLAG* | This study |
| RJD6513 | BY4741, pESC(HIS)-*P_GAL10_-RPL26A(4E)-FLAG* | This study |
| RJD6514 | BY4741 *rpl26a∆::KanMX4 rpl26b∆::KlLEU2*, pESC(HIS)-*P_GAL10_-RPL26A(4E)-FLAG* | This study |
| RJD6515 | BY4741 *rpl26a∆::KanMX4 rpl26b∆::KlLEU2 pdr5∆::KlURA3,* pESC(HIS)-*P_GAL10_-RPL26A-FLAG* | This study |
| RJD6516 | BY4741 *rpl26a∆::KanMX4 rpl26b∆::KlLEU2 pdr5∆::KlURA3,* pESC(HIS)-*P_GAL10_-RPL26A(4E)-FLAG* | This study |
| RJD6517 | BY4741 *pdr5∆::KanMX4*, pESC(HIS)-*P_GAL10_-RPL26A(4E)-FLAG* | This study |
| RJD6518 | BY4741 *tom1∆::KanMX4*, pESC(HIS)-*P_GAL10_-RPL26A(3E)-FLAG* | This study |
| RJD6519 | BY4741 *tom1∆::KanMX4*, pESC(HIS)-*P_GAL10_-RPL26A(4E)-FLAG* | This study |
| RJD6520 | BY4741 *KanMX6::P_RFA1_-3×HA-TOM1 pdr5∆::KlLEU2*, pESC(HIS)-*P_GAL10_-RPL26A(4E)-FLAG* | This study |
| RJD4781 | W303 *lys2∆::HIS3, arg4∆::KanMX4* | - |
| RJD6522 | W303 *lys2∆::HIS3, arg4∆::KanMX4 tom1∆::KlLEU2* | This study |
| RJD6523 | W303 *lys2∆::HIS3, arg4∆::KanMX4 tom1C3235A::KlLEU2* | This study |
| RJD6524 | BY4741 *acl4∆::KanMX4* | (Stelter et al., 2015) |
| RJD6525 | BY4741 *acl4∆::KanMX4 tom1C3235A::KlURA3* | This study |
| RJD6526 | W303 *URA3::P_ADH1_-AtTIR19myc* | This study |
| RJD6527 | W303 *URA3::P_ADH1_-AtTIR19myc tom1C3235A::KlLEU2* | This study |
| RJD6528 | W303 *URA3::P_ADH1_-AtTIR19myc HMO1-GFP-AID*::hyg* | This study |
| RJD6529 | W303 *URA3::P_ADH1_-AtTIR19myc HMO1-GFP-AID*::hyg tom1C3235A::KlLEU2* | This study |
| RJD6530 | W303 *URA3::P_ADH1_-AtTIR19myc Rrn3-GFP-AID*::hyg* | This study |
| RJD6531 | W303 *URA3::P_ADH1_-AtTIR19myc Rrn3-GFP-AID*::hyg tom1C3235A::KlLEU2* | This study |
| RJD6532 | W303 *URA3::P_ADH1_-AtTIR19myc KanMX6::P_RFA1_-9myc-AID*-RPA190* | This study |
| RJD6533 | W303 *URA3::P_ADH1_-AtTIR19myc KanMX6::P_RFA1_-9myc-AID*-RPA190 tom1C3235A::KlLEU2* | This study |
| RJD6428 | BY4741 *pdr5∆::KanMX4 tom1∆::KlLEU2* | This study |
| RJD6428 | BY4741 *pdr5∆::KanMX4 tom1∆::KlLEU2*, p*GAL1*-*RPL13B-HHZ** | This study |
| RJD6429 | BY4741 *pdr5∆::KanMX4 tom1∆::KlLEU2*, p*GAL1-RPL26A-HHZ** | This study |
| RJD6430 | BY4741 *pdr5∆::KanMX4 tom1∆::KlLEU2*, p*GAL1-RPL34A-HHZ** | This study |
| RJD6431 | BY4741 *pdr5∆::KanMX4 tom1∆::KlLEU2*, p*GAL1-RPL36A-HHZ** | This study |
| RJD6432 | BY4741 *pdr5∆::KanMX4 tom1∆::KlLEU2*, p*GAL1-RPS17B-HHZ** | This study |
| RJD6433 | BY4741 *pdr5∆::KanMX4 tom1∆::KlLEU2*, p*GAL1-RPS18A-HHZ** | This study |
| RJD6434 | BY4741 *pdr5∆::KanMX4 tom1∆::KlLEU2*, p*GAL1-RPS24A-HHZ** | This study |
| RJD6435 | BY4741 *pdr5∆::KanMX4 tom1∆::KlLEU2*, p*GAL1-RPS24B-HHZ** | This study |
| RJD6436 | BY4741 *pdr5∆::KanMX4 tom1∆::KlLEU2*, p*GAL1-HOG1-HHZ** | This study |
| RJD6437 | BY4741 *pdr5∆::KanMX4 tom1∆::KlLEU2*, p*GAL1-HHT2-HHZ** | This study |
| RJD6437 | BY4741 *pdr5∆::KanMX4*, p*GAL1-RPL8B-HHZ** | This study |
| RJD6437 | BY4741 *pdr5∆::KanMX4 tom1∆::KlLEU2*, p*GAL1-RPL8B-HHZ** | This study |
| RJD6525 | BY4741 *hmo1∆::KanMX4* | OBS* |
| RJD6525 | BY4741 *hmo1∆::KanMX4 tom1C3235A::KlURA3* | This study |
| RJD6459 | BY4741 *NOP56-RFP::KlURA3*, pESC(HIS3)-*P_GAL10_-RPL26A(4E)-GFP* | This study |
| RJD6656 | BY4741 *rps29a∆::KlLEU2* | This study |
| RJD6657 | BY4741 *tom1∆::KanMX4rps29a∆::KlLEU2* | This study |
| RJD2641 | BY4741 *hrd1∆::KanMX4* | OBS* |
| RJD2646 | BY4741 *san1∆::KanMX4* | OBS* |
| RJD2691 | BY4741 *ubr1∆::KanMX4* | OBS* |
| RJD4545 | BY4741 *doa10∆::KanMX4* | OBS* |
| RJD5400 | BY4741 *ltn1∆::KanMX4* | OBS* |
| RJD6708 | BY4741 *hrd1∆::KanMX4*, pESC(HIS3)-*P_GAL10_-RPL26A-FLAG* | This study |
| RJD6709 | BY4741 *san1∆::KanMX4*, pESC(HIS3)-*P_GAL10_-RPL26A-FLAG* | This study |
| RJD6710 | BY4741 *ubr1∆::KanMX4*, pESC(HIS3)-*P_GAL10_-RPL26A-FLAG* | This study |
| RJD6711 | BY4741 *doa10∆::KanMX4*, pESC(HIS3)-*P_GAL10_-RPL26A-FLAG* | This study |
| RJD6712 | BY4741 *ltn1∆::KanMX4*, pESC(HIS3)-*P_GAL10_-RPL26A-FLAG* | This study |
| RJD6713 | BY4741 *hrd1∆::KanMX4*, pESC(HIS3)-*P_GAL10_-RPL26A(4E)-FLAG* | This study |
| RJD6714 | BY4741 *san1∆::KanMX4*, pESC(HIS3)-*P_GAL10_-RPL26A(4E)-FLAG* | This study |
| RJD6715 | BY4741 *ubr1∆::KanMX4*, pESC(HIS3)-*P_GAL10_-RPL26A(4E)-FLAG* | This study |
| RJD6716 | BY4741 *doa10∆::KanMX4*, pESC(HIS3)-*P_GAL10_-RPL26A(4E)-FLAG* | This study |
| RJD6717 | BY4741 *ltn1∆::KanMX4*, pESC(HIS3)-*P_GAL10_-RPL26A(4E)-FLAG* | This study |
| RJD6718 | BY4741 *doa10∆::KanMX4*, pESC(HIS3)-*P_GAL10_-RPL26A(4E)-GFP* | This study |

OBS* : OpenBiosystems, yeast knockout collection

HHZ* : 6×His-HA-Protein A (ZZ domain)

**References**

Stelter, P., Huber, F.M., Kunze, R., Flemming, D., Hoelz, A., and Hurt, E. (2015). Coordinated Ribosomal L4 Protein Assembly into the Pre-Ribosome Is Regulated by Its Eukaryote-Specific Extension. Mol Cell *58*, 854-862.

Sung, M.K., Reitsma, J.M., Sweredoski, M.J., Hess, S., and Deshaies, R.J. (2016). Ribosomal proteins produced in excess are degraded by the ubiquitin-proteasome system. Mol Biol Cell.
