# Supplementary material for: A conserved quality-control pathway that mediates degradation of unassembled ribosomal proteins
Source: eLife. 2016 Aug 23;5:e19105. doi: 10.7554/eLife.19105 (PMC5026473; doi:10.7554/eLife.19105)
Supplement: Supplementary file 2. — DOI: http://dx.doi.org/10.7554/eLife.19105.023 [file elife-19105-supp2.docx]

**Table S2. Plasmids used in this study**

| **RDB** | **Plasmid** | **Source** |
| --- | --- | --- |
| 1564 | pCMV-HA-UBIQUITIN | (Kamitani et al., 1997) |
| 3112 | pGAL1-RPL26A-HHZ* | Open Biosystems |
| 3113 | pGAL1-RPL34A-HHZ* | Open Biosystems |
| 3114 | pGAL1-RPL36A-HHZ* | Open Biosystems |
| 3115 | pGAL1-RPL13B-HHZ* | Open Biosystems |
| 3116 | pGAL1-RPS18A-HHZ* | Open Biosystems |
| 3117 | pGAL1-RPS24A-HHZ* | Open Biosystems |
| 3118 | pGAL1-RPS24B-HHZ* | Open Biosystems |
| 3119 | pGAL1-RPS17B-HHZ* | Open Biosystems |
| 3120 | pGAL1-HOG1-HHZ* | Open Biosystems |
| 3121 | pGAL1-HHT2-HHZ* | Open Biosystems |
| 3122 | pESC (HIS) | Open Biosystems |
| 3123 | pESC (URA) | Open Biosystems |
| 3124 | pESC(HIS)-P_GAL10_-RPL26A-FLAG | (Sung et al., 2016) |
| 3125 | pESC(HIS)-P_GAL10_-RPL26A-GFP | (Sung et al., 2016) |
| 3126 | pRS314 | (Duncan et al., 2000) |
| 3127 | pRS314-TOM1 | (Duncan et al., 2000) |
| 3128 | pRS314-TOM1C3235A | (Duncan et al., 2000) |
| 3129 | pRS314-3xHA-TOM1 | (Duncan et al., 2000) |
| 3130 | pRS314-3xHA-TOM1C3235A | (Duncan et al., 2000) |
| 3132 | pESC(HIS)-P_GAL10_-RPL26A(4E)-FLAG | This study |
| 3133 | pESC(HIS)-P_GAL10_-RPL26A(4E)-GFP | This study |
| 3137 | pHyg–AID*–GFP | (Morawska and Ulrich, 2013) |
| 3140 | pKanMX6-P_RFA1_-9MYC-AID* | (Morawska and Ulrich, 2013) |
| 3141 | P_ADH1_-OsTIR1-9MYC | NBRP (pNHK53) |
| 3142 | pLKO-Tet-ON_shControl | (Thompson et al., 2014) |
| 3143 | pLKO-Tet-ON_shHUWE1 | (Thompson et al., 2014) |
| 3144 | P_CMV_-hRPL26-FLAG | Addgene (cat# 19972) |
| 3145 | pKlLEU2 | EUROSCARF (pUG73) |
| 3146 | pFA6a-GFP-KlURA3 | (Sung et al., 2008) |
| 3151 | pESC(HIS)-P_GAL10_-RPL26A(3E)-FLAG | This study |
| 3152 | pESC(HIS)-P_GAL10_-RPL26A(R12,13E)-FLAG | This study |
| 3153 | pESC(HIS)-P_GAL10_-RPL26A(R16E)-FLAG | This study |
| 3154 | pESC(HIS)-P_GAL10_-RPL26A(R27,28E)-FLAG | This study |
| 3155 | pESC(HIS)-P_GAL10_-RPL26A(R51,52E)-FLAG | This study |
| 3212 | pGAL1-RPL8B-HHZ | Open Biosystems |
| - | pcDNA3 | Invitrogen |
| - | pCDH-EF1-MCS-T2A-copMYCGFP | (Nguyen et al., 2012) |
| - | pGEX-6P-1 GST-*ct*Kap104^FL^ | Hoelz lab |
| - | pET28a His_6_-SUMO-*sc*Acl4^FL^ | Hoelz lab |
| - | pETDuet1 His_6_-SUMO-3xFLAG-*sc*RpL4^FL^ | Hoelz lab |
| - | pETDuet1 His_6_-SUMO-3xFLAG-*sc*RpL4^1-276^ | Hoelz lab |

HHZ* : 6×His-HA-Protein A (ZZ domain)

AID* : Auxin Inducible Degron

**References**

Duncan, K., Umen, J.G., and Guthrie, C. (2000). A putative ubiquitin ligase required for efficient mRNA export differentially affects hnRNP transport. Current Biology *10*, 687-696.

Kamitani, T., Kito, K., Nguyen, H.P., and Yeh, E.T.H. (1997). Characterization of NEDD8, a developmentally down-regulated ubiquitin-like protein. J Biol Chem *272*, 28557-28562.

Morawska, M., and Ulrich, H.D. (2013). An expanded tool kit for the auxin-inducible degron system in budding yeast. Yeast *30*, 341-351.

Nguyen, T.V., Angkasekwinai, P., Dou, H., Lin, F.M., Lu, L.S., Cheng, J.K., Chin, Y.E., Dong, C., and Yeh, E.T.H. (2012). SUMO-Specific Protease 1 Is Critical for Early Lymphoid Development through Regulation of STAT5 Activation. Mol Cell *45*, 210-221.

Sung, M.K., Ha, C.W., and Huh, W.K. (2008). A vector system for efficient and economical switching of C-terminal epitope tags in Saccharomyces cerevisiae. Yeast *25*, 301-311.

Sung, M.K., Reitsma, J.M., Sweredoski, M.J., Hess, S., and Deshaies, R.J. (2016). Ribosomal proteins produced in excess are degraded by the ubiquitin-proteasome system. Mol Biol Cell.

Thompson, J.W., Nagel, J., Hoving, S., Gerrits, B., Bauer, A., Thomas, J.R., Kirschner, M.W., Schirle, M., and Luchansky, S.J. (2014). Quantitative Lys-epsilon-Gly-Gly (diGly) Proteomics Coupled with Inducible RNAi Reveals Ubiquitin-mediated Proteolysis of DNA Damage-inducible Transcript 4 (DDIT4) by the E3 Ligase HUWE1. J Biol Chem *289*, 28942-28955.
